# Wheat Kernel Variety Identification Based on a Large Near-Infrared Spectral Dataset and a Novel Deep Learning-Based Feature Selection Method

**DOI:** 10.3389/fpls.2020.575810

**Published:** 2020-11-10

**Authors:** Lei Zhou, Chu Zhang, Mohamed Farag Taha, Xinhua Wei, Yong He, Zhengjun Qiu, Yufei Liu

**Affiliations:** ^1^College of Biosystems Engineering and Food Science, Zhejiang University, Hangzhou, China; ^2^Key Laboratory of Spectroscopy Sensing, Ministry of Agriculture and Rural Affairs, Hangzhou, China; ^3^Synergistic Innovation Center of Jiangsu Modern Agricultural Equipment and Technology, Zhenjiang, China; ^4^School of Agricultural Equipment Engineering, Jiangsu University, Zhenjiang, China

**Keywords:** NIR hyperspectroscopy, wheat kernel classification, feature selection, convolutional neural network, attention mechanism

## Abstract

Near-infrared (NIR) hyperspectroscopy becomes an emerging nondestructive sensing technology for inspection of crop seeds. A large spectral dataset of more than 140,000 wheat kernels in 30 varieties was prepared for classification. Feature selection is a critical segment in large spectral data analysis. A novel convolutional neural network-based feature selector (CNN-FS) was proposed to screen out deeply target-related spectral channels. A convolutional neural network with attention (CNN-ATT) framework was designed for one-dimension data classification. Popular machine learning models including support vector machine (SVM) and partial least square discrimination analysis were used as the benchmark classifiers. Features selected by conventional feature selection algorithms were considered for comparison. Results showed that the designed CNN-ATT produced a higher performance than the compared classifier. The proposed CNN-FS found a subset of features, which made a better representation of raw dataset than conventional selectors did. The CNN-ATT achieved an accuracy of 93.01% using the full spectra and keep its high precision (90.20%) by training on the 60-channel features obtained via the CNN-FS method. The proposed methods have great potential for handling the analyzing tasks on other large spectral datasets. The proposed feature selection structure can be extended to design other new model-based selectors. The combination of NIR hyperspectroscopic technology and the proposed models has great potential for automatic nondestructive classification of single wheat kernels.

## Introduction

Wheat is one of the most important agricultural products. Various varieties of wheat are cultivated to adapt to different planting environments and to improve the yield and quality. Different varieties of wheat kernels have different characters and values. The purity of wheat kernels is of great concern by breeders, planters, and consumers (Ebrahimi et al., 2014). Wheat kernels of different variety share some similar characteristics, which makes it difficult to distinguish with the naked eye. Mass spectrometry-based methods have been widely accepted for inspection of wheat quality owing to their high sensitivity (Koistinen and Hanhineva, 2017). However, they are destructive methods, and expensive instrument is required. Recent advances in machine learning, computer vision, and spectroscopic sensing promote a series of nondestructive testing techniques for crop seeds evaluation (Sabanci et al., 2017; Ding et al., 2019; Xia et al., 2019; Fabiyi et al., 2020). Hyperspectral imaging (HSI) is an emerging tool with the advantages of collecting spectral and spatial information simultaneously. The obtained data is in the shape of a hypercube (width ^∗^ height ^∗^ number of channels). Each spatial pixel is a spectral vector. It allows a user to collect data of many samples by scanning a single HSI image. Therefore, it is very suitable for analyzing large quantities of crop kernels (Feng et al., 2019).

The digital information collected by hyperspectroscopic instruments are always in large volume. It contains a lot of redundant information and causes troubles for data analysis. Feature selection becomes a critical procedure in the pretreatment of high-dimensional spectral data (Lin et al., 2012; Zhang et al., 2016). Feature selection methods are expected to improve the performance of classification/regression models by screening out a subset of informative features and to accelerate the model training procedure as well. Another way for compressing the volume of a dataset is feature extraction.

Research efforts are attracted in the field of feature selection in spectral data processing (Balabin and Smirnov, 2011; Lei and Sun, 2020). Some methods aim at selecting the most important subset by univariate statistical tests, such as univariate feature selection (UFS) (Emura et al., 2019). This kind of methods operate with a high efficiency. However, as a fact, the feature selectors based on checking the amount of information in each channel only use the features, excluding corresponding labels. It is possible to screen out the features, which are not highly related to the final target. Similar problems were discussed in an article about feature extraction (Yuan et al., 2018). Some methods search the important features by training a linear machine learning model. The coefficients of the trained models are considered as the importance scores of each feature. A typical example is support vector machine (SVM) feature selector (SVM-FS) (Khaled et al., 2018; Pes, 2019). The model-based feature selectors consider the target labels. However, the quality of the selected features depends on the capacity of the machine learning model. It is hard to find a subset of effective features by a low-performance model. The model-based feature selection methods have great potential to screen out informative and output-related features by improving the used model. There are also several tree-based feature selection approaches, such as extra-trees classifier (ETC) (Geurts et al., 2006; Krol and Polanska, 2017) and random forest (RF) (Khaled et al., 2018).

Concerning large dataset processing, there are some limitations in traditional modeling methods. Deep learning algorithms become emerging tools to solve the complex modeling tasks. Different deep architectures consisting of nonlinear processing units have been introduced for seed variety identification based on spectral datasets. Ozkan et al. (2019) designed a convolutional neural network (CNN) for variety discrimination of wheat grain. Spectral images of the grains were collected by multiple spectroscopic sensors to make a dataset. It was found that the CNN method could identify the category of the grains based on a spectral image with hundreds of grains. The recognition of each individual grain was not involved. Several researchers investigated the application of deep learning algorithms on kernel-level analysis of crop seeds. Qiu et al. (2018) proposed a CNN classifier for rice seed variety identification using NIR spectroscopy. The CNN model showed its superiority in the classification task, achieving a higher precision than the compared K nearest neighbors (KNN) and SVM. Zhu et al. (2019b) applied CNN methods to discriminate three varieties of soybean seeds by processing NIR spectral data. A satisfactory result was achieved.

According to the surveyed articles above, deep learning classifiers performed better than conventional ones in spectra pattern recognition. In general, a deep learning model is a combination of linear/nonlinear data processing layers with different operation rules. Different structures of the layers or the whole networks can be custom defined to implement different applications. The existing deep learning applications involve classification, regression, feature extraction, objective detection, and so on (Zhang et al., 2018). The application of CNN architecture was also extended to variable selection (Liu et al., 2019), which calculated the importance score according to weights of the first convolutional layer. Very few of the abovementioned articles about seed classification used a very large dataset to evaluate the models. The deep learning methods can show its advantages more prominently in big datasets. In this study, a novel CNN-based feature selection algorithm was proposed for searching the informative spectral channels beneficial to the final classification problem. A large NIR spectral dataset of 30 varieties of wheat kernel (147,096 kernels in total) was prepared for experiment. A CNN model with attention mechanism was designed to process the selected features. Several well-known feature selection methods including UFS, SVM-FS, ETC, and classification methods including RBF-SVC, partial least squares discrimination analysis (PLSDA) were employed as benchmark methods. The selected features were visualized, and the classification accuracies were compared.

## Materials and Methods

### Wheat Kernel Preparation

Thirty varieties of wheat kernels harvested in 2019 were collected from a local seed company in Shuyang, Jiangsu Province, China. The wheat kernels were stored under the same condition after harvest (dried, packaged by woven plastic bags, and delivered to the laboratory). The wheat plants were grown in the same field, and the kernels were harvested in the same year. Based on the time-sequence, the kernels were taken out of the package and sent for analysis, without any physical or chemical operation used on the kernels as preprocessing. Indeed, there were some differences among all the kernel in shape, weight, water content, and so on. A large number of samples of each variety were scanned to build the dataset, which was expected to provide adequate knowledge for the deep learning models. The category number, wheat variety, and number of samples are listed in Table 1.

**TABLE 1:** Overview of the dataset properties.

No.	Variety name	Abbreviation	Number of samples (Cal/Val/Pre*)
1	aikang58	AK58	2,400/1,200/1,054
2	bainong207	BN207	2,400/1,200/1,431
3	bainong4199	BN4199	2,400/1,200/1,332
4	bainong889	BN889	2,400/1,200/1,335
5	baomai218	BM218	2,400/1,200/1,259
6	baomai330	BM330	2,400/1,200/1,277
7	baomai5	BM5	2,400/12,00/1,355
8	fengdecunmai12	FDCM12	2,400/1,200/1,353
9	fengdecunmai20	FDCM20	2,400/1,200/1,360
10	guanmai1	GM1	2,400/1,200/1,361
11	huaimai20	HM20	2,400/1,200/1,283
12	huaimai40	HM40	2,400/1,200/1,273
13	huaimai41	HM41	2,400/1,200/1,280
14	huaimai920	HM920	2,400/1,200/1,134
15	jiangmai816	JM816	2,400/1,200/1,356
16	jiangmai919	JM919	2,400/1,200/1,356
17	jimai211	JM211	2,400/1,200/1,353
18	jimai22	JM22	2,400/1,200/1,241
19	lunxuan99	LX99	2,400/1,200/1,285
20	luomai9	LM9	2,400/1,200/1,357
21	ruihuamai518	RHM518	2,400/1,200/1,346
22	saidemai1	SDM1	2,400/1,200/1,354
23	tiechuaifu99	TCAF99	2,400/1,200/1,353
24	weilong169	WL169	2,400/1,200/1,204
25	xinong20	XN20	2,400/1,200/1,342
26	xinong979	XN979	2,400/1,200/1,350
27	xumai36	XM36	2,400/1,200/1,356
28	xumai818	XM818	2,400/1,200/1,354
29	yannong19	YN19	2,400/1,200/1,279
30	yunong035	YN035	2,400/1,200/1,123
Total			72,000/24,000/39,096

*Cal/Val/Pre means the calibration (Cal), validation (Val), and prediction (Pre) sets.

### Near-Infrared Hyperspectral Image Scanning

Spectral images of the wheat kernels were collected by an NIR hyperspectral system with the spectral range from 874 to 1,734 nm. The main components included a spectrograph, a camera with lens, tungsten halogen light source, and a conveyer belt driven by a stepper motor for line scan. The detail information and the operation process of the sensing system were the same as the descriptions in Qiu et al. (2018). In this research, the moving speed of the conveyer belt, the exposure time of the camera, and the distance from the lens to the plate were set as 8.7 mm/s, 3 ms, and 20 cm, respectively.

**FIGURE 1:**
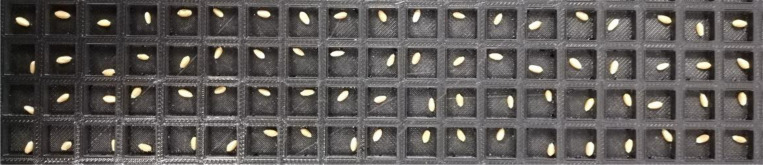
A batch of wheat kernels.

The kernels were placed in a plate with grid for spectral image acquisition (see Figure 1). The plate was made of special materials that generate a very low reflectance in near-infrared range. A total of 147,096 wheat kernels were sampled. The samples of each variety were randomly separated into training, validation, and prediction set. In each variety, 2,400 kernels were used for calibration, 1,200 kernels were for validation, and the rest was for prediction. Details are listed in Table 1.

### Spectra Extraction and Preprocessing

The collected spectral image should be corrected by black and white calibration first:

(1) $$S_{c}=\frac{S_{raw}-S_{dark}}{S_{white}-S_{dark}}$$

The dark reference *S*_*dark*_ was obtained by covering the camera lens with opaque cap, and the white reference *S*_*white*_ was obtained by scanning a white Teflon tile. *S*_*raw*_ is the raw spectral data, and *S*_*c*_ is the dark-white calibrated data.

**FIGURE 2:**
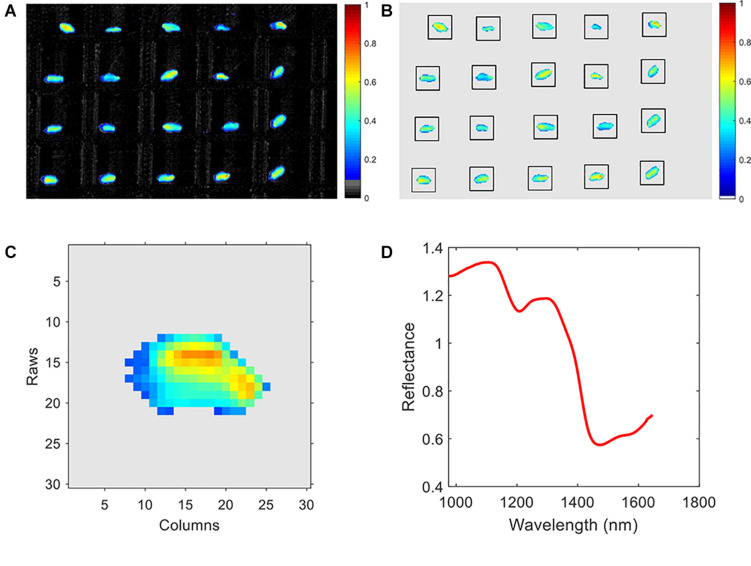
Spectral data pre-processing. **(A)** Pseudo-image of the raw spectral image with background, **(B)** pseudo-image of the spectral image without background, **(C)** pseudo-image of an individual wheat kernel, and **(D)** the mean spectra of the wheat kernel in **(C)**.

**FIGURE 3:**
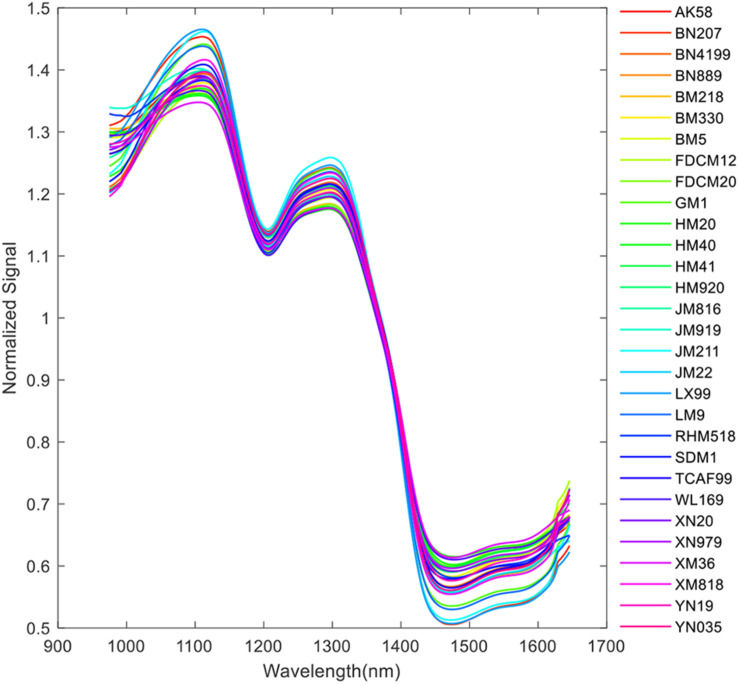
Spectral profiles of the 30 varieties of wheat kernels.

Due to the high-level noises at the start and end of the band range, only the range of 975–1,645 nm (200 bands in total) was selected in this research. The pseudo-image of the 20th band is shown in Figure 2A. An adaptive threshold segmentation method was used to remove the background and get the region of interest (ROI). The adaptive threshold was calculated by Otsu method (Otsu, 1979). Morphology erosion and dilation operations (a filter in disk shape with diameter of 5 pixels) were adopted for removing the small noisy pixels. The connected component labeling method was used to detect the region of each kernel in the image. Due to the use of the plate shown in Figure 1, the process of background removing and kernel region segmentation becomes very easy. Then, a square region with side length of 30 pixels for each kernel was extracted. The pseudo-image of ROI of the 20th band is shown in Figure 2B. Thus, the information of each kernel was recorded in a hypercube (width: 30 pixels, height: 30 pixels, channels: 200). Take one kernel for example; the pseudo-image of the 20th band is shown in Figure 2C. The average spectra of each individual hypercube was calculated and then processed by mean filter (window size of 5) and average normalization. The normalized spectra were used for representing the sample for further analysis. As an example, the normalized average spectra of the wheat kernel in Figure 2C is shown in Figure 2D. The pre-processed average spectra of all 30 different varieties of wheat kernels are shown in Figure 3.

### Conventional Feature Selection Methods and Classifiers

#### Feature Selection Methods

Univariate feature selection (UFS) is a simple approach, which evaluates the importance of features by univariate statistical tests. For the data of classification purpose, Chi-square test was commonly used (Pes, 2019). Tree-based methods are also a choice for feature selection. These models calculate impurity-based importance. The importance value in the trained tree could be regarded as the importance score of each individual feature (Khaled et al., 2018). Linear estimators with sparse penalty term can be used as feature selectors. Due to the L1 norm term added in the loss function, some of the coefficients are fixed as 0. The features with nonzero coefficients are considered as key features. As typical examples, the least absolute shrinkage and selection operator (LASSO) method (Kok et al., 2019; Chakraborty et al., 2020) is commonly used in regression problems. Logistic regression and linear SVM are adopted in classification problems (Pes, 2019). The selected features are further processed by another model. UFS with Chi-square test, ETC, and linear SVM were used in this study. These three methods were implemented by using scikit-learn^1^, a very popular machine learning tool kit for Python.

#### Classifiers

Radial basis function support vector machine classifier (RBF-SVC) is a popular pattern recognition method (Burges, 1998). Due to its high capacity of handling nonlinearity, RBF-SVC has been widely used in spectral data classification (Jimenezcarvelo et al., 2019). In the RBF-SVC model, penalty coefficient C and the kernel parameter gamma should be optimized to realize high performances. The optimized value of C and gamma in RBF-SVC models were determined by grid search strategy and checking the validation accuracies. PLSDA is another widely accepted linear classification algorithm. It can be realized by modifying partial least squares regression (PLSR) method (Lee et al., 2018). In PLSDA model, the number of the principle component (comp) was optimized by checking the precision on the validation set. RBF-SVC and PLSDA, a nonlinear and a linear classifier, were selected as the benchmark classifiers in this research.

### The Proposed Convolutional Neural Network Architectures for Feature Selection and Classification

**FIGURE 4:**
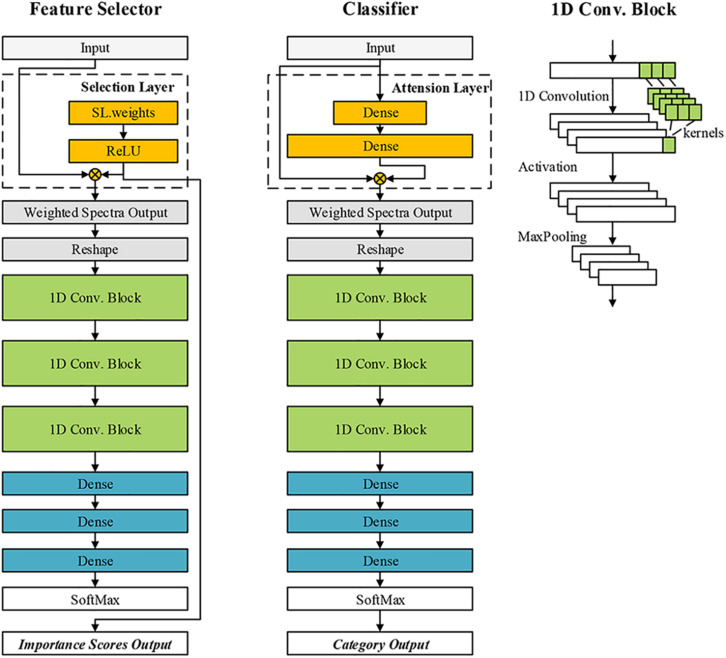
The architectures of the proposed convolutional neural network (CNN)-based models for classification and feature selection.

CNN-based models have become very important machine intelligence algorithms for big data analysis. These models were popular in computer vision for RGB image processing (Ou et al., 2020). The applications of CNN were also extended to one-dimension data (such as pixel-level spectra) (Zhou et al., 2019) and three-dimension data (Zhang et al., 2018; Mehrkanoon, 2019). In this research, the features of the wheat kernel were in a shape of 200^∗^1. A one-dimension CNN architecture with attention mechanism was presented for classification. Another CNN model with feature selection block was designed for optimal band selection. We define them as CNN-ATT and CNN-FS, respectively. The architectures of the designed networks are shown in Figure 4. The custom blocks were annotated by a dashed box. The CNN-ATT and CNN-FS shared a similar structure. First, the input data with a shape of N^∗^Ch is processed by a custom block. N denotes batch size, and Ch is the number of feature channels. The output of the custom block was the weighted spectra vector. The weighted spectra vector should be reshaped to N^∗^1^∗^Ch for convolution operation. Then, the reshaped data was processed by three one-dimension convolution (1D Conv.) blocks (the number of the kernels, the kernel size, the strides were set as 16, 3, 1, respectively) and three dense layers (the number of neurons in three dense layers were 512, 128, 30, respectively). The last part was the SoftMax layer. The ELU activation (Qiu et al., 2018) was used in the networks (excluding the activation function in the two custom blocks). The MaxPooling layer (pool size and stride are 2, 2, respectively) batch normalization operation was used after each convolution layer.

#### CNN-FS

As for the CNN-FS, the custom block was defined as the selection block (SL block). Referring to the idea of LASSO algorithm (Lee and Cai, 2020), our aim was to calculate a vector of scores to indicate the importance of each channel on the target classification task. The weights of this block (WSL) were generated as the same shape as the input. A nonlinear activation function was used to process the WSL. The output of the SL block was defined as:

(2) $$\begin{array}{c} Y_{SL}=f_{A}\left(W_{SL}\right)\cdot X\\ W_{SL}=\left[W_{1},W_{2},\ldots ,W_{Ch}\right],X=\left[X_{1},X_{2},\ldots ,X_{Ch}\right] \end{array}$$

where ⋅ denotes multiplication of corresponding elements in the two matrixes. *W*_*SL*_ is the weights of the SL block, *Y*_*SL*_ is the weighted spectra, and *f*_*A*_ is the activation function. Define score_*SL*_ = *f*_*A*_(*W*_*SL*_) as the importance scores. Here, some alternative activation functions could be SoftMax activation, Sigmoid, and ReLU. However, SoftMax makes the scores too small, which is unfavorable for model training. Although Sigmoid function can map the values to (0,1), it is also not suitable here. The target was to remove some non-informative channels and tune the scores of these channels to 0. If the Sigmoid was used, the Wi for a non-informative channel should be tuned close to negative infinity to get a score of 0 (or a value smaller than −4 to get a score close to 0). ReLU function could add some nonlinearity to the SL block and ensure the scores are all positive. Thus, ReLU was finally employed:

(3) $$\text{ReLU}\left(a\right)=\left\{ \begin{array}{cc} a & a>0\\ 0 & a\leq 0 \end{array}\right.$$

In this block, random initialization not suitable for the SL block. The WSL was initialized as [1,1,…,1], and the activated *W*_*SL*_ (or the initial scores) was also [1,1,…,1], which controls the initial importance scores all equal for each channel.

Then, the output of the SL block was reshaped and calculated by a one-dimension CNN classifier. The feature selection model was trained to minimize the classification error and keep the value of *f*_*A*_(*W*_*SL*_) small. The loss function is defined as follows:

(4) $$Loss\left(y,\tilde{y}\right)=\frac{1}{N}\sum_{i=1}^{S}\left(-\sum_{j=1}^{q}y_{j}^{i}\log\left({\tilde{y}}_{j}^{i}\right)\right)+\lambda \sum_{j=1}^{q}\left(\text{ReLU}\left(\left|W_{j}\right|\right)\right)$$

The loss function consisted of two terms. The first part was cross entropy loss, which controls the classification precision. The second part was the sum of the activated weights in the SL block, which led the scores of unimportant features close to zero.

#### CNN-ATT

As for the CNN-ATT, the custom block was defined as attention block (ATT block). Attention mechanism enabled the network to pay more attention to some specific regions of the input data and weaken the focus on other regions. The attention scores were calculated by encoding the input via two dense layers:

(5) $$Y_{ATT}=f_{2}\left(W_{ATT}^{\left(2\right)}\cdot f_{1}\left(W_{ATT}^{\left(1\right)}\cdot X+b_{1}\right)+b_{2}\right)\cdot X$$

where the symbol “⋅” denotes matrix multiplication, *W*_*ATT*_ is the weights of the ATT block, and *Y*_*ATT*_ is the weighted spectra by attention. Define ${\text{score}}_{ATT}=f_{2}\left(W_{ATT}^{\left(2\right)}\cdot f_{1}\left(W_{ATT}^{\left(1\right)}\cdot X+b_{1}\right)+b_{2}\right)$ as the attention scores.

The number of the neurons in the second dense layer should be equal to the dimension of the input, while that in the first dense layer could be set by developers. Although both the ATT block and SL block could generate a vector of scores, the functions of them differed. In the trained model, score_*SL*_is constant, while the value of score_*ATT*_ varies with different *X*. The score_*ATT*_ cannot adopted for feature selection, but it can be effective for building the classifier. The CNN-based models were programmed base on the MXNET framework^2^.

## Results and Discussion

### Training Procedures for the Convolutional Neural Network-Based Feature Selector and Convolutional Neural Network With Attention

**FIGURE 5:**
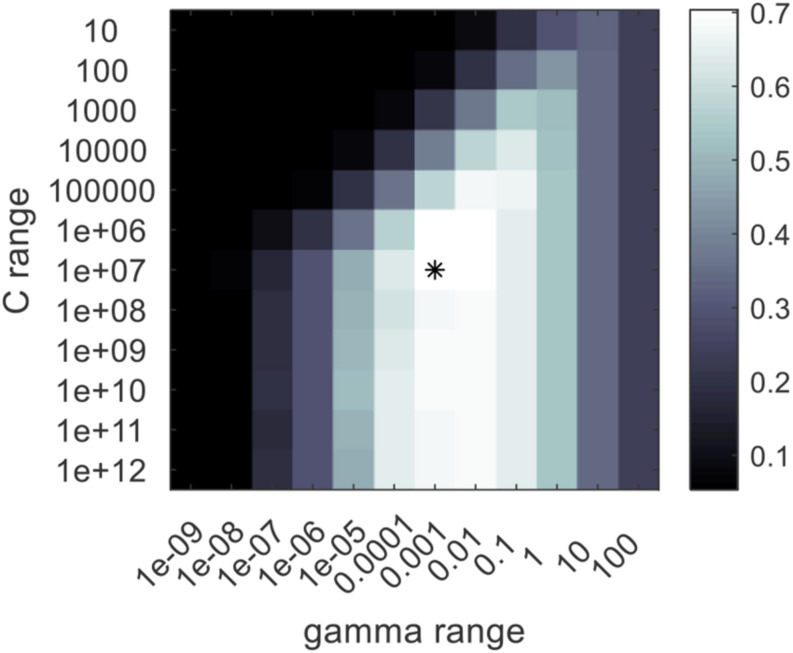
The grid search result for the optimization of the radial basis function support vector machine classifier (RBF-SVC) model. The best combination of the RBF-SVC parameters is marked by “*”.

In this study, the calibration dataset was used for training the models, the validation set was only for model adjustment, and the independent prediction set was for performance evaluation of the models. The methods for constructing conventional feature selectors and classifiers are described in the Conventional Feature Selection Methods and Classifiers section. The searching ranges were set as 10^1^–10^12^ and 10^–9^–10^2^. The searching range for comp was set from 1 to the number of the input features. The optimized parameters of PLSDA models and RBF-SVC models are listed in Table 2. The result of grid-search (take the modeling based on full spectra as an example) is shown in Figure 5; the best combination of the parameters is marked by “^∗^.”

**TABLE 2:** The classification accuracies achieved by different feature selectors and classifiers.

Methods	Features selectors	Selected channels	Parameters*	Calibration	Validation	Prediction
Partial least squares discrimination analysis (PLSDA)	/	200	comp = 200	0.7008	0.6937	0.6880
	Convolutional neural network-based feature selector (CNN-FS)	60	comp = 60	0.5980	0.5895	0.5942
	Support vector machine-feature selector (SVM-FS)	60	comp = 60	0.5133	0.5040	0.5075
	Univariate feature selection (UFS)	60	comp = 60	0.4213	0.4183	0.4132
	Extra-trees classifier (ETC)	60	comp = 60	0.5531	0.5474	0.5488
Radial basis function support vector machine classifier (RBF-SVC)	/	200	C = 10^7^, g = 0.001	0.8706	0.8594	0.8599
	CNN-FS	60	C = 10^6^, g = 0.01	0.8668	0.8555	0.8572
	SVM-FS	60	C = 10^7^, g = 0.01	0.7603	0.7433	0.7516
	UFS	60	C = 10^7^, g = 0.01	0.6590	0.6488	0.6487
	ETC	60	C = 10^6^, g = 0.01	0.8267	0.8123	0.8167
CNN-ATT	/	200	nn = 64	***0.9686***	***0.9303***	***0.9301***
	CNN-FS	60	nn = 32	***0.9428***	***0.9012***	***0.9020***
	SVM-FS	60	nn = 32	0.8924	0.8438	0.8394
	UFS	60	nn = 32	0.7778	0.7399	0.7420
	ETC	60	nn = 32	0.9233	0.8742	0.8773

*The used hyperparameters of different models. “comp” is the number of principle components for PLSDA. “C” and “g” are the C and gamma value for SVM. “nn” is the number of neurons in the first dense layer of the attention block. The bold and italic values are the best classification results achieved by modeling on full spectra and 60 selected features, respectively.

The CNN-ATT model was trained by minimizing the output of the cross-entropy loss using the Adam algorithm with a dynamic learning rate. The loss function used for CNN-ATT was the cross-entropy loss, while that for CNN-FS is defined in Eq. 4. The hyper parameters in CNN-based models were adjusted by repeated training for many times and observing the accuracy of the validation set. A group of parameters was found for obtaining a stable model. The number of epochs was set as 500. The value of λ in Eq. 4 was optimized as 0.1. We used a scheduled learning rate, the initial value was 0.0005, the period was defined as 100, and learning rate decay was set as 0.1. In other words, the learning rate was 0.0005 in the first 100 epochs, and it was decreased to 0.00005 in the next 100 epochs, and so on. At the start of the training, a relatively large learning rate was used to speed up training, and smaller values were used with the gradual convergence of the model. With the mentioned training procedure, the CNN models produced a low and stable loss value and a high classification accuracy on validation set.

For training the CNN-FS, the custom loss function defined in Eq. 4 was utilized. Other properties of the trainer were configured as the same as that for the mentioned CNN classifier. Each of the feature selectors provides a vector of importance scores. Theoretically, the more features, the better the modeling effect. In order to better compare the effect of feature selector and ensure the calculation efficiency, we chose the features of the highest 60 scores for study. This value could be changed according to the requirement of practical applications.

### Feature Selection Results

**FIGURE 6:**
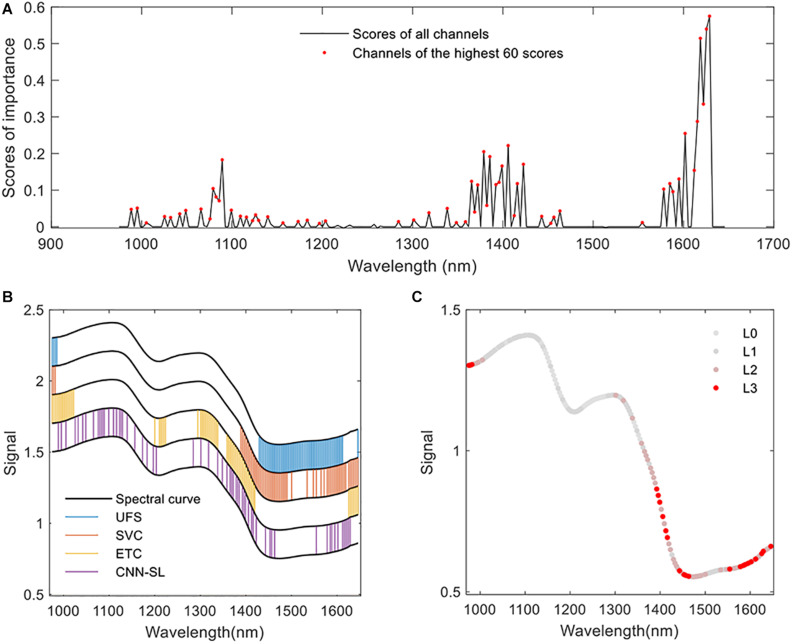
The feature selection results. **(A)** The scores of each feature channels calculated by CNN-based feature selector (CNN-FS). **(B)** Feature selected by different algorithms. The black curves are produced by adding different offsets (0.2, 0.4, 0.6, 0.8, 1), which is only used for the convenience of visualization. The values of the y axis have no special meaning. **(C)** The overall result of feature selection. “L3” means that three of the four selectors find that the specific channel is important, “L2” means that two of them find the importance, and so on. The closer the color of the channel is to red, the more selectors select the channel.

The results of the feature selection procedure are visualized in Figure 6. In Figure 6A, the scores calculated by the proposed CNN-FS are shown. The channel was given a nonzero score, which was found important for the target classification problem. The scores of non-informative channels were adjusted close to zero. In Figure 6B, the locations of the selected bands by different methods are marked. The UFS method found that the important bands lie in the range of 975–985 and 1,429–1,612 nm. The SVC method selected a range of 975–982, 1,389–1,500 and some bands in 1,534–1,646 nm. The ETC methods produced high importance score at the range of 975–1,022, 1,200–1,224, 1,294–1,338, 1,359–1,419, and 1,625–1,648 nm. The channels found by the abovementioned methods gathered in several continuous ranges. The channels selected by CNN-FS scattered in the whole range of 975–1,645 nm. To further analyze the common parts of the selected features, an indicator of each channel was calculated to indicate how many selectors decide that the channel was important (see Figure 6C). In Figure 6C, “L0” means that no selector found that the corresponding channel is important, “L1” means that one selector found the importance in the corresponding channel, and so on. The common ranges were centered at about 978, 1,405, and 1,601 nm.

### Classification Result Analysis

The effects of feature selectors based on UFS, ETC, linear SVC, and CNN were compared. The classification accuracies reached by different classifiers with different feature extraction algorithms are summarized in Table 2. PLSDA is a recognized method for spectral analysis; however, the structure of the linear PLS model is simple. In this study, it is hard to predict the probability distribution of each category based on such a large and complex dataset. The PLSDA-based methods achieved lowest accuracy rates (0.4132∼0.6880). The RBF-SVC method did better than the PLSDA. The CNN-ATT classifier realized higher accuracies on prediction set than those achieved by any other classifiers considered in this study. The highest classification accuracy of 0.9301 was realized by CNN-ATT with full spectra. The structures of the RBF-SVC and CNN were more complex than PLS. They could handle the dataset with high nonlinearity. Thus, the performances of these models were better than PLSDA.

Take the PLSDA classifiers as examples; the accuracies vary from 0.4132 to 0.6880. Modeling based on the UFS selected features performed the worst. The PLSDA models calibrated by SVM-FS and ETC produced better prediction results, and the model built based on features selected by CNN-FS showed the highest precision. The same phenomenon could be found in other classifiers. The feature channels selected by the proposed CNN-based feature extractor significantly improved the classification rate for all the tested classifiers. Furthermore, with 60 optimal bands screened by CNN-FS, the CNN-ATT classifier still achieved an accuracy of 0.902. The performance was not significantly decreased after feature selection and still kept high than 0.9, which proves the effectiveness/rationality of the features selected by the proposed CNN-FS. It can be inferred that CNN-based feature selectors could find the small differences lying in those selected channels, which was crucial to the classification task. The precision reached by CNN-ATT with other compared selectors was lower than 0.9 (0.7420∼0.8773). Experiments showed the superiority of the proposed CNN-ATT classifier and the CNN-FS feature selector.

**FIGURE 7:**
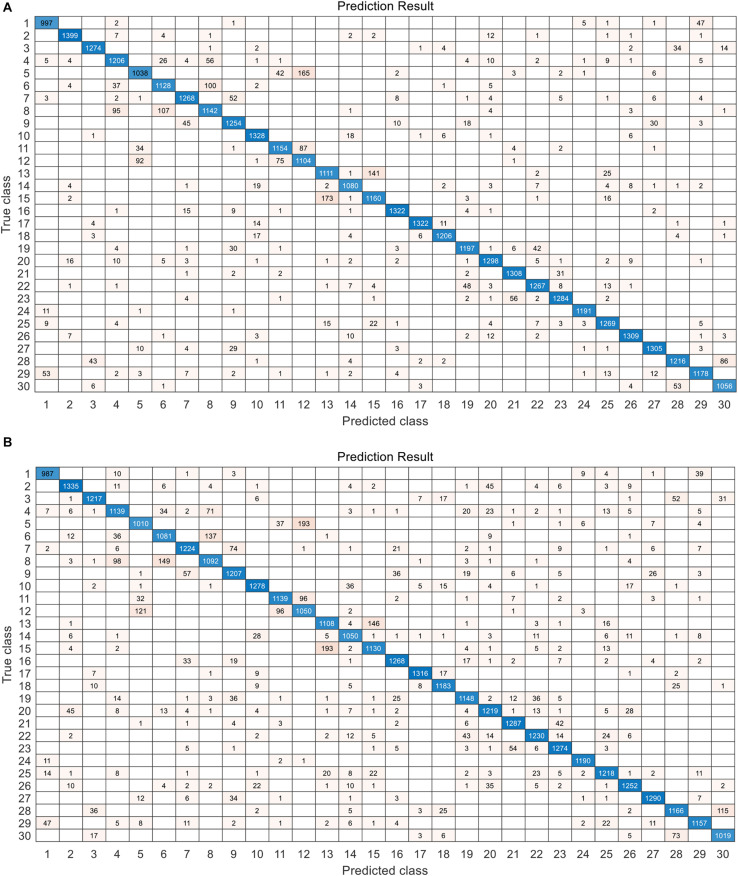
The confusion map of the best classification result on the prediction set. **(A)** The result achieved by CNN with attention (CNN-ATT) model with 200 features. **(B)** The result achieved by CNN-ATT model with 60 features selected by CNN-FS.

The confusion maps of the best results (on the prediction set) achieved by the full spectra and the selected features are shown in Figure 7. Relatively high error rate was in some categories. For example, the samples of BM218 (category “5”) were misjudged as HM40 (category “12”). The highest error rate in a single variety was about 20%. The low error was about 1%. The distribution of error rate was approximately the same in the two experiments (the full spectra-based modeling and 60 feature-based modeling).

## Discussion

NIR spectrometry has some advantages toward other technologies for processing a large number of kernels. Mass spectrometry could analyze the biochemical information of crop kernels with high sensitivity (Sorensen et al., 2002; Wadood et al., 2020). However, it is destructive, requiring high time consumption and relatively high cost. Crop seed classification applications based on electronic nose and thermal imaging were limited by the requirement of environment condition, and those based on X-ray imaging was also limited due to the high cost (Rahman and Cho, 2016). In addition, varieties of portable NIR spectral sensors (Crocombe, 2018; Deidda et al., 2019) with tiny size and low cost are available on the market, which makes the NIR spectroscopy-based technologies become promising tools for crop kernel inspection in practical application the future industry. Potential applications include breeding, seed adulteration inspection, and so on.

Feature selection methods choose the most informative features in the raw dataset. It is critical to big data analysis and data mining. The existing feature selector can be divided into two types, unsupervised feature selection and supervised. As for the former, univariate statistical tests is used on each feature channel to calculate the importance. As for the latter, a classification or regression model is trained. The coefficients of the model are considered as the importance of the features, such as SVM-FS. Also, some supervised methods remove the non-informative feature during the training, such as uninformative variable elimination partial least square (UVE-PLS) (Wang et al., 2012). The supervised feature selector can also be considered as a model-based selector. According to the results in Table 2, the PLSDA methods could not discriminate the samples with high accuracy. The reliability of the features selected by low-performance models could not be guaranteed. Similar linear models could realize satisfactory performance in relatively small datasets. For example, in the article (Zhu et al., 2019a), the LR classifier calibrated by original features and CNN-extracted features reached similar accuracies of those achieved by CNN classifiers. However, PLSDA was insufficient to build a high-performance estimator on such a large and complex dataset in this study. The methods that are popular in small dataset processing may not be suitable for applications on large and complex datasets. Thus, some common methods such as SPA-PLS, UVE-PLS, and the methods based on loading weights of latent variables of PLS (Li et al., 2007, 2019; Zhang et al., 2017, 2020; Li and Hui, 2019) were not evaluated in this study. The UFS is a typical unsupervised feature selector. The performances of the classifiers did even worse based on such non-output-related selection method. The UFS tested weak for valid information identification. The CNN-based classifiers performed better than the SVM-based methods, and the classification results achieved by features from the CNN-FS was better than those from the SVM-FS. Thus, the capability of the core model is essential to the model-based feature selectors. As for the deep learning-based selector in Liu et al. (2019), the weights of the kernels in the first convolution layer was used to indicate the importance of the feature. As a fact, the 1D convolution kernel operates on the adjacent region centered on the target feature channel (rather than operates on the single target channel). Thus, the trained weights were affected by adjacent feature channels. The importance scores, which were determined by calculating the sum of weights in each convolutional kernel in the first convolutional layer, might be disturbed. Also, the scores of the unimportant channels were nonzero values in the trained model (Liu et al., 2019). These nonzero weighted features could still make a certain impact on classification.

The proposed CNN-FS used a powerful deep learning model to find the deep relation between the input and the target output. A custom linear selection layer was added for feature ranking. Each weight of the selection layer controls an individual feature, and the custom-defined loss function controls the adjustment process of the coefficients in the selection layer. The scores of non-informative channels could be tuned close to zero, as shown in Figure 6A. All of the three evaluated classifiers learned better knowledge from the selected features of CNN-FS than those from other feature selectors. The efficiency of the proposed CNN-FS was confirmed by the experimental results. This research provided an approach that adds a linear part at the start of the model to make the nonlinear model applicable for feature selection. In the future work, this idea can also be extended to develop other nonlinear feature selectors.

## Conclusion

A large NIR spectral dataset of wheat kernels was collected for a 30-category classification task. The CNN-FS method was proposed for screening out the key bands of spectral data. Moreover, a CNN classifier with attention mechanism was designed for wheat kernel identification. The CNN-ATT method produced a higher precision than that realized by PLSDA and RBF-SVC. The selected key spectral channels from the CNN-FS acted more informative than those found by the compared existing feature selection methods. The CNN-ATT model achieved an accuracy of 0.9301 on the prediction set using full spectra, and it kept a high performance (accuracy of 0.9020) for classification when using 60 key channels selected by CNN-FS. The CNN-FS method was proved to be suitable for feature selection on the large dataset.

## Data Availability Statement

The datasets presented in this study can be found in online repositories. The names of the repository/repositories and accession number(s) can be found in the article/Supplementary Material.

## Author Contributions

LZ was responsible for the conceptualization, methodology, software, data curation, and writing – original draft preparation. CZ was also responsible for the conceptualization, writing – reviewing and editing, and funding acquisition. MT was responsible for data curation. XW was in charge of the resources and funding acquisition. YH was responsible for the project administration and writing – reviewing and editing. ZQ supervised and wrote – reviewing and editing, and acquired the funds. YL was responsible for the investigation, resources, writing – reviewing and editing, visualization, and funding acquisition. All authors contributed to the article and approved the submitted version.

## Conflict of Interest

The authors declare that the research was conducted in the absence of any commercial or financial relationships that could be construed as a potential conflict of interest.

## Footnotes

1 https://scikit-learn.org/

2 http://mxnet.incubator.apache.org/
