# Supplementary figures and images for: Wheat Kernel Variety Identification Based on a Large Near-Infrared Spectral Dataset and a Novel Deep Learning-Based Feature Selection Method
Source: Front Plant Sci. 2020 Nov 10;11:575810. doi: 10.3389/fpls.2020.575810 (PMC7683420; doi:10.3389/fpls.2020.575810)

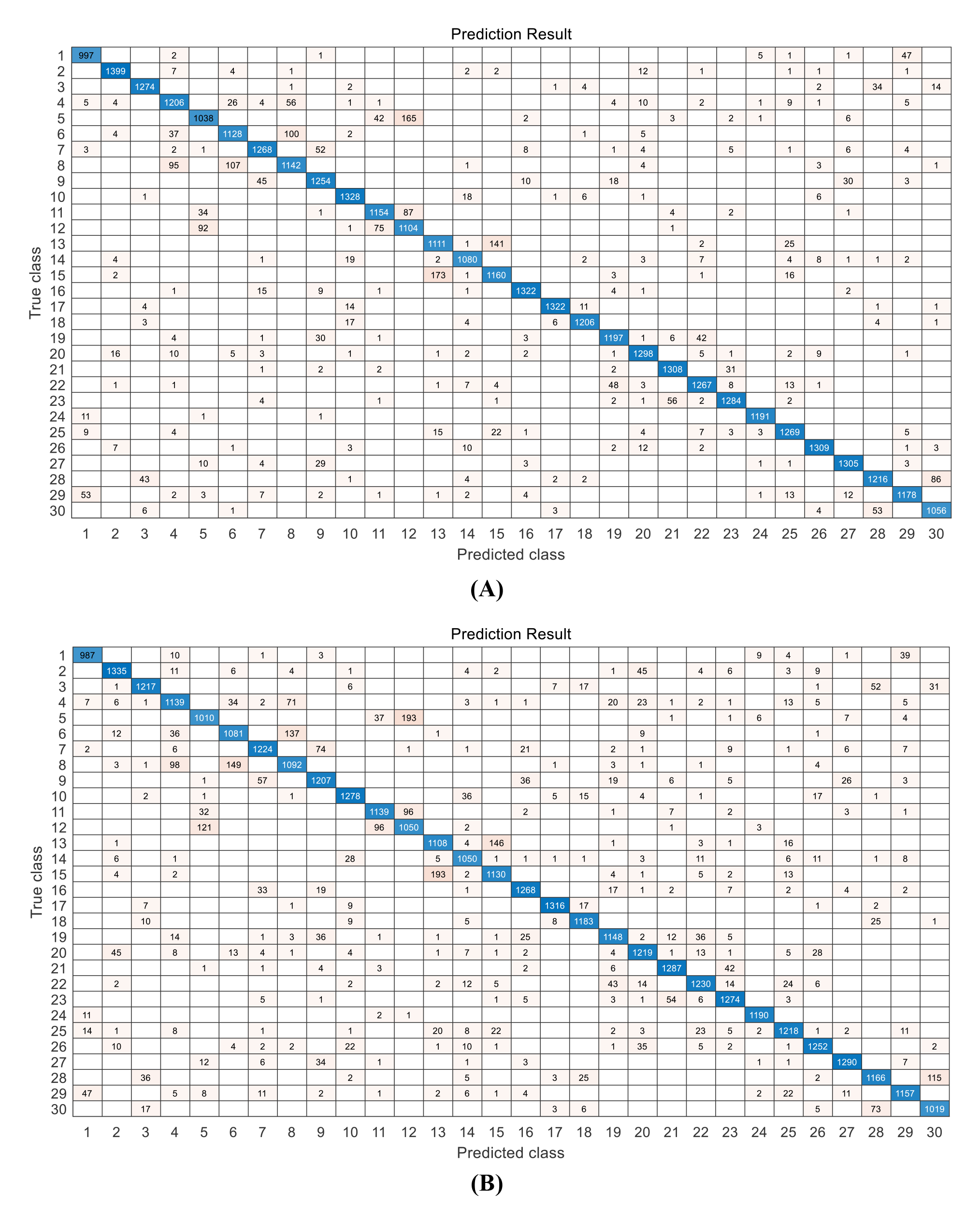

Supplement: Supplementary file 1 [file Data_Sheet_1.ZIP › Figure7.tif]

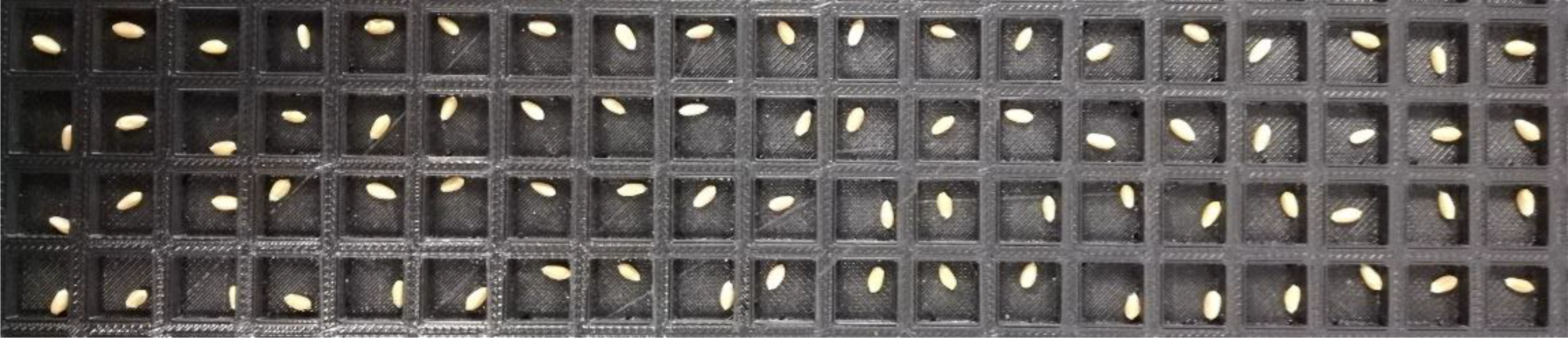

Supplement: Supplementary file 1 [file Data_Sheet_1.ZIP › Figure1.tif]

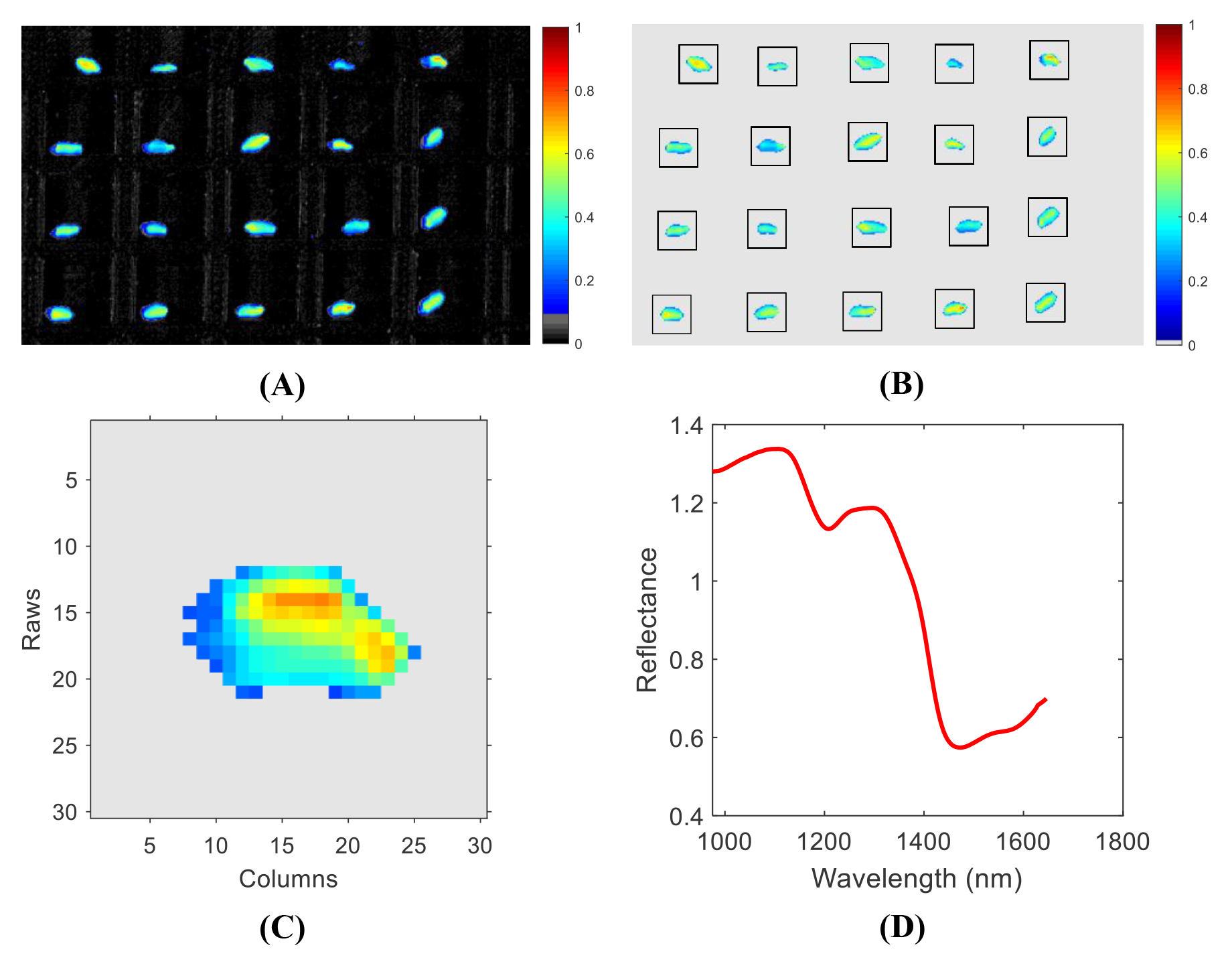

Supplement: Supplementary file 1 [file Data_Sheet_1.ZIP › Figure2.tif]

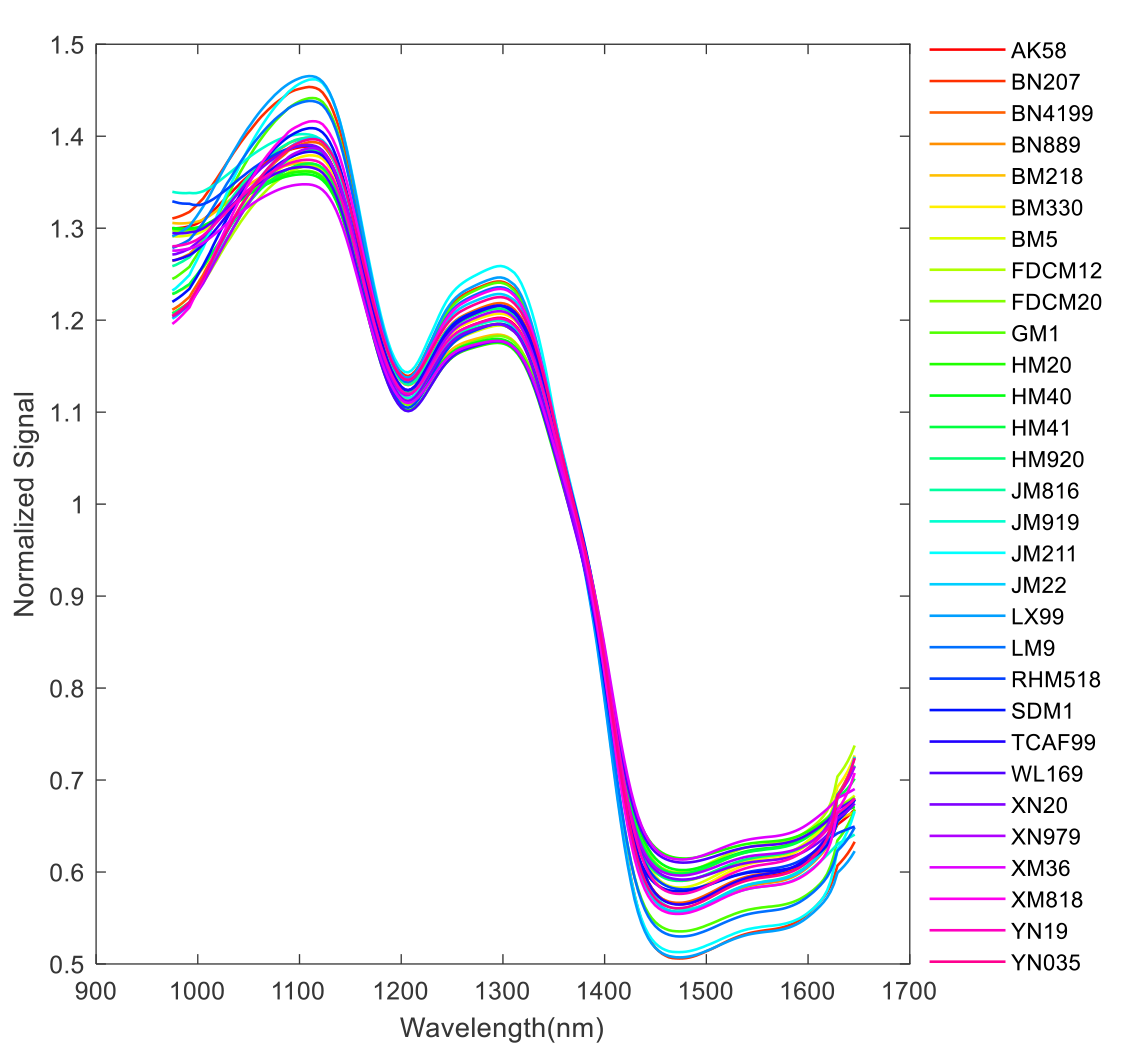

Supplement: Supplementary file 1 [file Data_Sheet_1.ZIP › Figure3.tif]

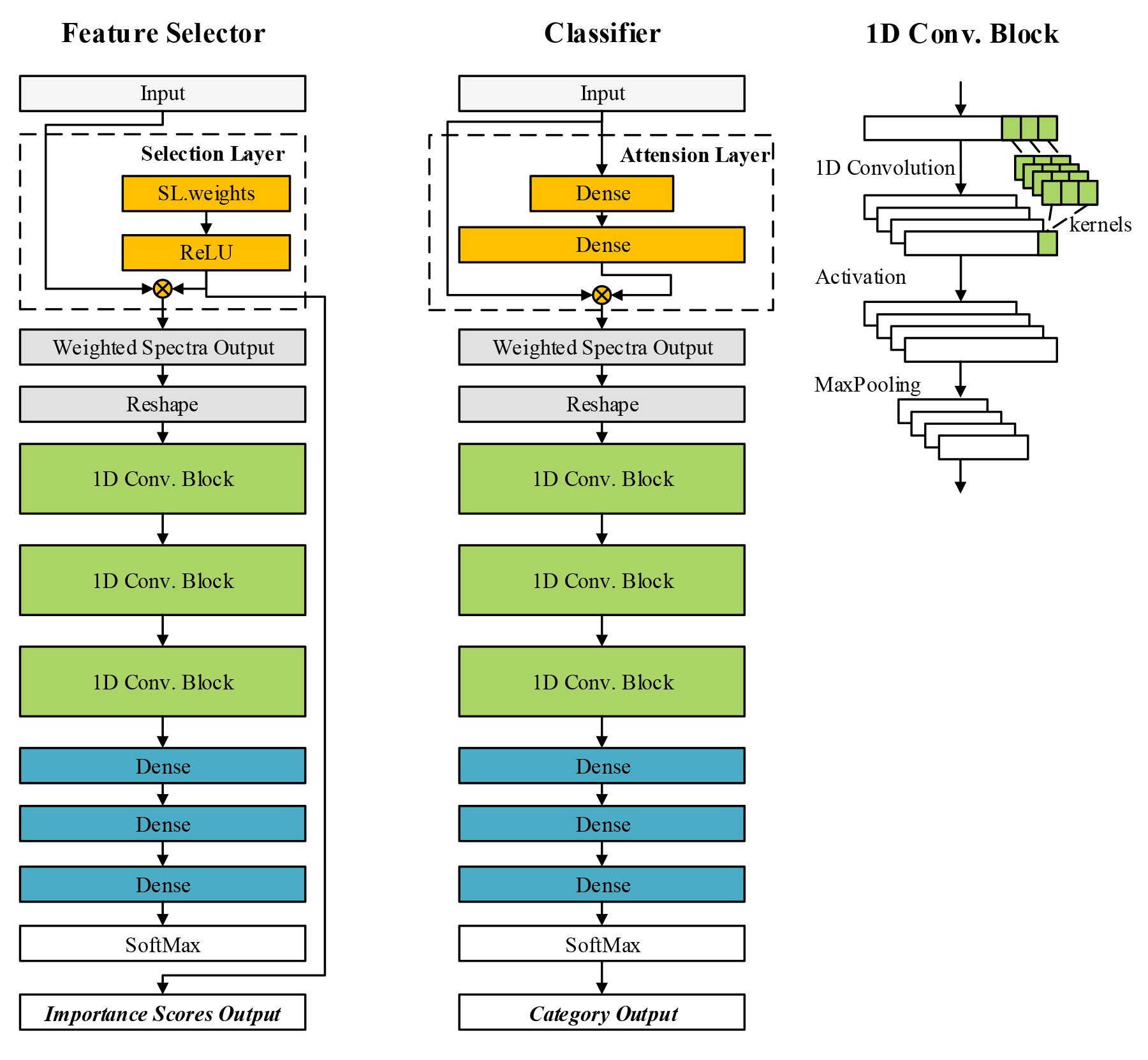

Supplement: Supplementary file 1 [file Data_Sheet_1.ZIP › Figure4.tif]

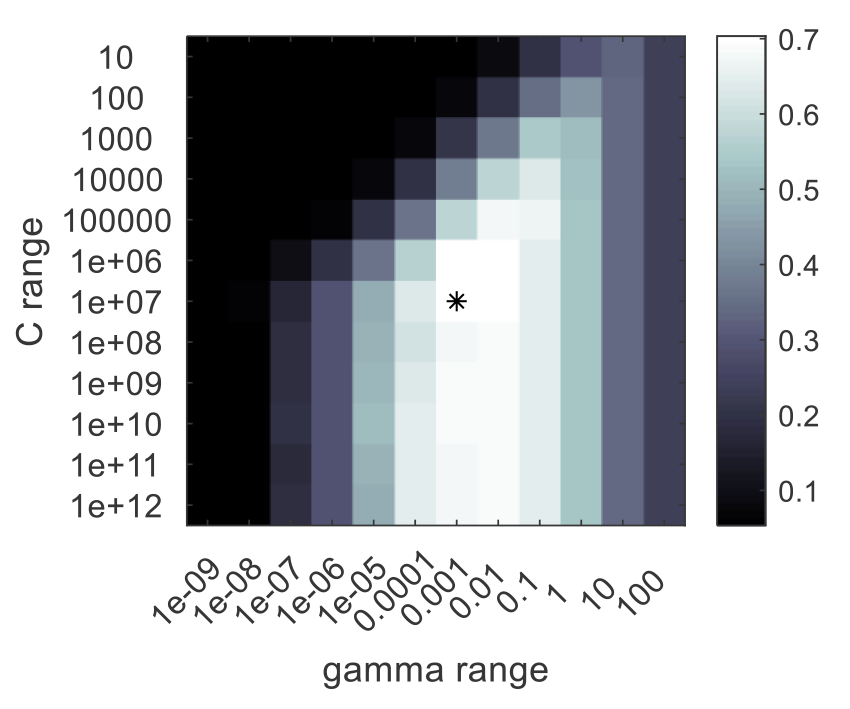

Supplement: Supplementary file 1 [file Data_Sheet_1.ZIP › Figure5.tif]

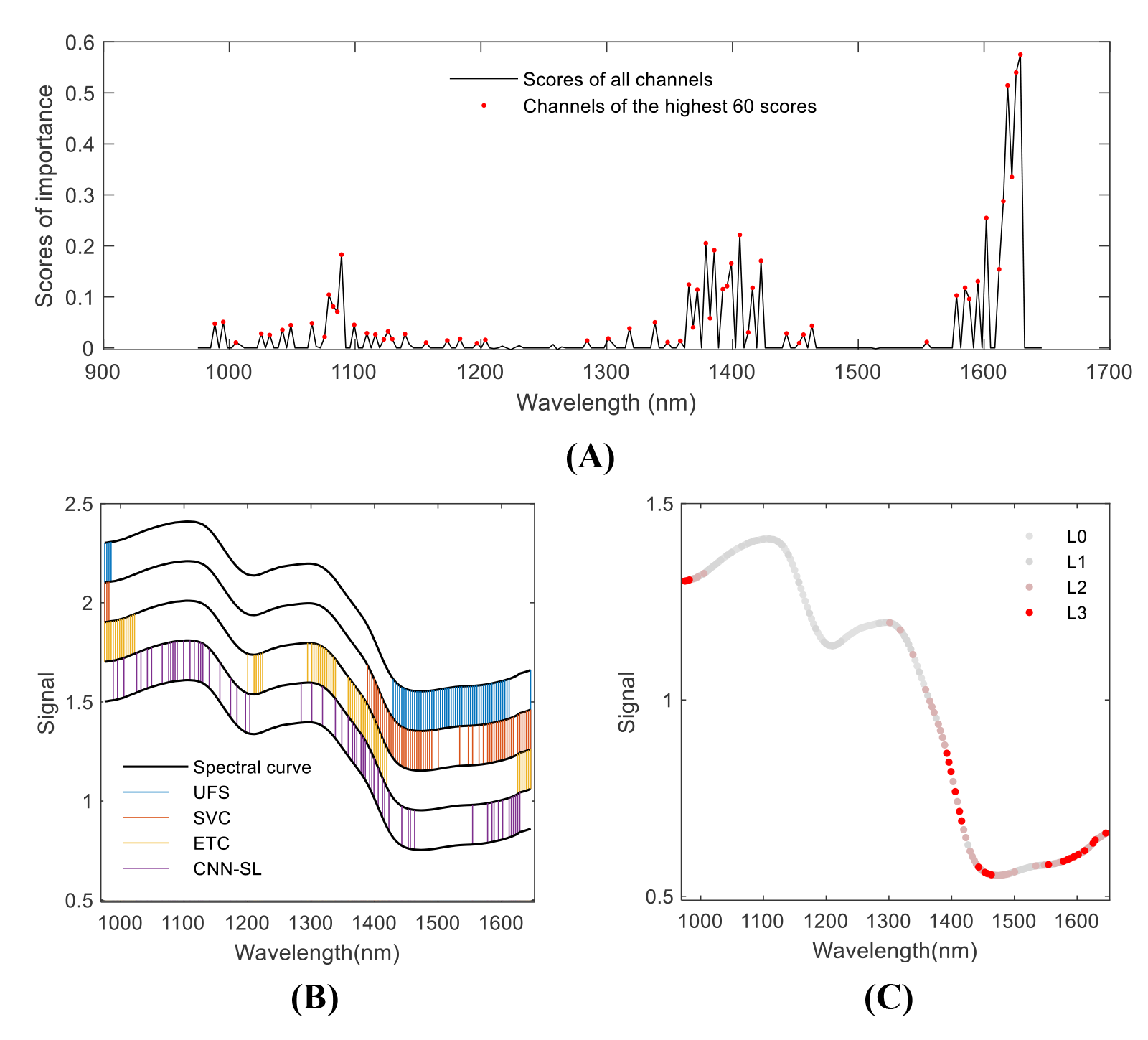

Supplement: Supplementary file 1 [file Data_Sheet_1.ZIP › Figure6.tif]
